# Supplementary material for: Identification and dynamics of the human ZDHHC16-ZDHHC6 palmitoylation cascade
Source: eLife. 2017 Aug 15;6:e27826. doi: 10.7554/eLife.27826 (PMC5582869; doi:10.7554/eLife.27826)
Supplement: Supplementary file 1. — (A) Model reactions. The model of DHHC6 palmitoylation contains 35 different reactions, describing synthesis, folding, degradation, and the enzymatic reactions of DHHC6 palmitoylation/depalmitoylation. In the following table we describe in detail how the rates for those reactions are calculated. (B) Mass balance equations. The following table describes the mass balance for each of the species of DHHC6 model. The rates of the mass balance of each state are described in detail in A. (C) Model parameters. The output of GA is a set of optimal solutions, where a solution is a complete set of parameter needed to perform model simulations. From this set we extracted a sub-set of 152 solutions that obtained a GA score better than a set threshold for each objective. During the analysis the model was simulated for each set of parameters of the sub-set. We then reported in this paper the mean of the outputs along with the first and third quartile of their distribution. [file elife-27826-supp1.docx]

**Supplementary file 1A. Model reactions.** The model of DHHC6 palmitoylation contains 35 different reactions, describing synthesis, folding, degradation, and the enzymatic reactions of DHHC6 palmitoylation/depalmitoylation. In the following table we describe in detail how the rates for those reactions are calculated.

|  | | |
| --- | --- | --- |
| Reaction | **Forward rate** | **Reverse rate** |
| 1. Synthesis and folding |  |  |
|  | *V1=* | *-* |
|  | *V2=* | *-* |
| 1. Palmitoylation |  |  |
|  | *V3=* | *V4=* |
|  | *V5=* | *V6=* |
|  | *V7=* | *V8=* |
|  | *V9=* | *V10=* |
|  | *V11=* | *V12=* |
|  | *V13=* | *V14=* |
|  | *V15=* | *V16=* |
|  | *V17=* | *V18=* |
|  | *V19=* | *V20=* |
|  | *V21=* | *V22=* |
|  | *V23=* | *V24=* |
|  | *V25=* | *V26=* |
| 1. Degradation |  |  |
|  | *V27=* |  |
|  | *V28=* |  |
|  | *V29=* |  |
|  | *V30=* |  |
|  | *V31=* |  |
|  | *V32=* |  |
|  | *V33=* |  |
|  | *V34=* |  |
|  | *V35=* |  |

Where the competition terms $f$ and $b$ in S1 Table are defined as:

| $f_{1}$ |  |
| --- | --- |
| $f_{2}$ |  |
| $f_{3}$ |  |
| $b_{1}$ |  |
| $b_{2}$ |  |
| $b_{3}$ |  |

for each palmitoylation and depalmitoylation step we define the kinetic parameters of the reaction as:

$$\begin{matrix} V_{f1}=k_{catP16c1}\cdot DHHC_{16t} \\ Km_{f1}=\frac{k_{c1P16ub}+k_{catP16c1}}{k_{c1P16b}} \\ V_{b1}=k_{catAPTc1}\cdot APT_{2t} \\ Km_{f1}=\frac{k_{c1APTub}+k_{catAPTc1}}{k_{c1APTb}} \end{matrix}$$

where $k_{catP16c1}$ is the catalytic constant for DHHC16 when catalyzing palmitoylation of C^100^ on DHHC6, while $k_{catAPTc1}$ is the catalytic constant for APT2 for the same site. $k_{c1P16b}$ and $k_{c1P16ub}$ are the binding and unbinding rate constants of DHHC6 to DHHC16 on site C^100^ respectively. $k_{c1APTb}$and $k_{c1APTub}$k_c1APTub_ are the binding and unbinding rate constants of DHHC6 to APT1 on site C^100^ respectively. In our model we assume that the binding and unbinding rate to DHHC6 and APT can be different for each state of the model.

**Supplementary file 1B. Mass balance equations**. The following table describes the mass balance for each of the species of DHHC6 model. The rates of the mass balance of each state are described in detail in S1 Table.

| Mass balance | Mass balance |
| --- | --- |
| $\frac{dU}{dt}=v1-v2-v27$ | $\frac{dC^{110}}{dt}=v9+v17+v22-v10-v18-v21-v32$ |
| $\frac{dC^{000}}{dt}=v2+v4+v6+v8-v3-v5-v7-v28$ | $\frac{dC^{101}}{dt}=v11+v13+v24-v12-v14-v23-v33$ |
| $\frac{dC^{100}}{dt}=v3+v10+v12-v4-v9-v11-v29$ | $\frac{dC^{011}}{dt}=v15+v19+v26-v16-v20-v25-v34$ |
| $\frac{dC^{010}}{dt}=v7+v18+v20-v8-v17-v19-v30$ | $\frac{dC^{111}}{dt}=v21+v23+v25-v22-v26-v24-v35$ |
| $\frac{dC^{001}}{dt}=v5+v14+v16-v6-v13-v15-v31$ |  |

**Supplementary file 1C. Model parameters.** The output of GA is a set of optimal solutions, where a solution is a complete set of parameter needed to perform model simulations. From this set we extracted a sub-set of 152 solutions that obtained a GA score better than a set threshold for each objective. During the analysis the model was simulated for each set of parameters of the sub-set. We then reported in this paper the mean of the outputs along with the 1^st^ and 3^rd^ quartile of their distribution.

| Parameter | Median Value | Range |
| --- | --- | --- |
| $vs$ [C]a/[T]^b^ | 3.00$\cdot10^{-3}$ | [2.59$\cdot10^{-3}$-3.31$\cdot10^{-3}$] |
| $kf$ 1/[T] | 4.20$\cdot10^{-1}$ | [2.98$\cdot10^{-1}$-6.69$\cdot10^{-1}$] |
| $V_{f1}$ [C]/[T] | 6.03 | [4.54-6.92] |
| $Km_{f1}$ [C] | 2.02$\cdot10^{-2}$ | [1.62$\cdot10^{-2}$-2.48$\cdot10^{-2}$] |
| $V_{f2}$ [C]/[T] | 4.21$\cdot10^{-4}$ | [1.18$\cdot10^{-4}$-9.89$\cdot10^{-4}$] |
| $Km_{f2}$ [C] | 1.33$\cdot10^{-5}$ | [9.40$\cdot10^{-6}$-2.16$\cdot10^{-5}$] |
| $V_{f3}$ [C]/[T] | 8.07$\cdot10^{-2}$ | [4.30$\cdot10^{-2}$-1.49$\cdot10^{-1}$] |
| $Km_{f3}$ [C] | 1.06$\cdot10^{-2}$ | [8.21$\cdot10^{-3}$-1.22$\cdot10^{-2}$] |
| $V_{b3}$ [C]/[T] | 8.12$\cdot10^{-3}$ | [5.07$\cdot10^{-3}$-1.13$\cdot10^{-2}$] |
| $Km_{b3}$ [C] | 1.63$\cdot10^{-3}$ | [7.99$\cdot10^{-4}$-3.22$\cdot10^{-3}$] |
| $V_{b2}$ [C]/[T] | 5.86$\cdot10^{-2}$ | [4.52$\cdot10^{-2}$-7.44$\cdot10^{-2}$] |
| $Km_{b2}$ [C] | 1.74$\cdot10^{-2}$ | [1.18$\cdot10^{-2}$-2.29$\cdot10^{-2}$] |
| $V_{b1}$ [C]/[T] | 1.18$\cdot10^{-1}$ | [9.04$\cdot10^{-2}$-1.42$\cdot10^{-1}$] |
| $Km_{b1}$ [C] | 3.71$\cdot10^{-3}$ | [2.27$\cdot10^{-3}$-6.13$\cdot10^{-3}$] |
| $kd_{m}$ 1/[T] | 2.56$\cdot10^{-1}$ | [2.16$\cdot10^{-1}$-3.50$\cdot10^{-1}$] |
| $kd_{C^{000}}$1/[T] | 1.80$\cdot10^{-2}$ | [1.47$\cdot10^{-2}$-2.72$\cdot10^{-2}$] |
| $kd_{C^{100}}$1/[T] | 1.33$\cdot10^{-1}$ | [8.81$\cdot10^{-2}$-1.97$\cdot10^{-1}$] |
| $kd_{C^{010}}$1/[T] | 4.82$\cdot10^{-3}$ | [1.58$\cdot10^{-3}$-1.32$\cdot10^{-2}$] |
| $kd_{C^{001}}$1/[T] | 3.89$\cdot10^{-2}$ | [2.07$\cdot10^{-2}$-6.04$\cdot10^{-2}$] |
| $kd_{C^{110}}$1/[T] | 1.28$\cdot10^{-1}$ | [3.66$\cdot10^{-2}$-2.50$\cdot10^{-1}$] |
| $kd_{C^{101}}$1/[T] | 5.59$\cdot10^{-2}$ | [2.74$\cdot10^{-2}$-2.41$\cdot10^{-1}$] |
| $kd_{C^{011}}$1/[T] | 3.42$\cdot10^{-4}$ | [6.46$\cdot10^{-5}$-1.36$\cdot10^{-3}$] |
| $kd_{C^{111}}$1/[T] | 2.32 | [7.14$\cdot10^{-1}$-7.31] |
| $V_{f12}$ [C]/[T] | 6.43$\cdot10^{-2}$ | [3.36$\cdot10^{-2}$-1.07$\cdot10^{-1}$] |
| $V_{f13}$ [C]/[T] | 6.04$\cdot10^{-1}$ | [1.54$\cdot10^{-1}$-1.36] |
| $V_{f23}$ [C]/[T] | 2.89 | [1.58-4.74] |
| $V_{f123}$ [C]/[T] | 5.61$\cdot10^{-1}$ | [1.37$\cdot10^{-1}$-2.43] |
| $V_{b12}$ [C]/[T] | 1.83 | [7.00$\cdot10^{-1}$-5.64] |
| $V_{b13}$ [C]/[T] | 1.40 | [5.01$\cdot10^{-1}$-5.28] |
| $V_{b23}$ [C]/[T] | 9.49$\cdot10^{-2}$ | [5.60$\cdot10^{-2}$-1.54$\cdot10^{-1}$] |
| $V_{b123}$ [C]/[T] | 9.35$\cdot10^{-1}$ | [2.09$\cdot10^{-1}$-4.19] |
| $DHHC_{16t}$ [C] | 1.73$\cdot10^{-1}$ | [1.05$\cdot10^{-1}$-3.10$\cdot10^{-1}$] |
| $APT_{2t}$ [C] | 1.85$\cdot10^{-1}$ | [1.42$\cdot10^{-1}$-2.20$\cdot10^{-1}$] |
