# Supplementary material for: Identification and dynamics of the human ZDHHC16-ZDHHC6 palmitoylation cascade
Source: eLife. 2017 Aug 15;6:e27826. doi: 10.7554/eLife.27826 (PMC5582869; doi:10.7554/eLife.27826)
Supplement: Supplementary file 2. — (A) Half-life of DHHC6 in different palmitoylation states. The half-life was estimated from the decay rate constant obtained through parameter estimation. The half-life is calculate as: ln(2)/kd. (B) Total amount of protein in steady state relative to WT for WT and CAA mutant in different conditions. The table shows the total protein in steady state in the model relative to the abundance of DHHC6 observed in steady state in WT conditions. Simulations are performed for WT and CAA mutant under control conditions, after overexpression of DHHC16 and after silencing of APT2. [file elife-27826-supp2.docx]

**Supplementary file 2A. Half-life of DHHC6 in different palmitoylation states.** The half-life was estimated from the decay rate constant obtained through parameter estimation. The half-life is calculate as: ln(2)/kd.

**Supplementary file 2B. Total amount of protein in steady state relative to WT for WT and CAA mutant in different conditions.** The table shows the total protein in steady state in the model relative to the abundance of DHHC6 observed in steady state in WT conditions. Simulations are performed for WT and CAA mutant under control conditions, after overexpression of DHHC16 and after silencing of APT2.
