# Supplementary material for: Identification and dynamics of the human ZDHHC16-ZDHHC6 palmitoylation cascade
Source: eLife. 2017 Aug 15;6:e27826. doi: 10.7554/eLife.27826 (PMC5582869; doi:10.7554/eLife.27826)
Supplement: Supplementary file 3. — (A) Parameters used for stochastic simulations. The following parameters were obtained through the conversion of the deterministic parameters estimated by the GA (see Dallavilla et al., 2016). (B) Stoichiometric matrix used for stochastic simulations. In the following table we define the stoichiometry and the directionality of the reactions of the model. Each reaction has a directionality that define which are the reagents and which are the products. In this table each line represents a model reaction, while in the columns we find all the states of the model. For each reaction the states of the model that take part to the reaction as reagents are marked with −1, while the states that participate as products are marked with 1. The matrix that is formed in this way allow to attribute the correct directionality to model reactions during the calculation of the mass balance for each state of the model. (C) Propensity function used for stochastic simulations. In the first column of the table, each line describes a reaction of the model. To each reaction is associated a rate, in the second column, that describes the probability of that reaction to happen at each time step of the stochastic simulation. [file elife-27826-supp3.docx]

**Supplementary file 3A. Parameters used for stochastic simulations.** The following parameters were obtained through the conversion of the deterministic parameters estimated by the GA (see {Dallavilla, 2016 #211}).

| Parameter | Value | Units |
| --- | --- | --- |
| $vs$ | 8.76$\cdot10^{1}$ | molecules/h |
| $kf$ | 4.20$\cdot10^{-1}$ | 1/h |
| $V_{f1}$ | 1.76$\cdot10^{5}$ | molecules/h |
| $Km_{f1}$ | 5.91$\cdot10^{2}$ | Molecules |
| $V_{f2}$ | 1.23$\cdot10^{1}$ | molecules/h |
| $Km_{f2}$ | 3.89$\cdot10^{-1}$ | Molecules |
| $V_{f3}$ | 2.36$\cdot10^{3}$ | molecules/h |
| $Km_{f3}$ | 3.09$\cdot10^{2}$ | molecules |
| $V_{b3}$ | 1.58$\cdot10^{2}$ | molecules/h |
| $Km_{b3}$ | 4.77$\cdot10^{1}$ | Molecules |
| $V_{b2}$ | 1.71$\cdot10^{3}$ | molecules/h |
| $Km_{b2}$ | 5.07$\cdot10^{2}$ | Molecules |
| $V_{b1}$ | 3.46$\cdot10^{3}$ | molecules/h |
| $Km_{b1}$ | 1.08$\cdot10^{2}$ | Molecules |
| $kd_{m}$ | 2.56$\cdot10^{-1}$ | 1/h |
| $kd_{C^{000}}$ | 1.80$\cdot10^{-2}$ | 1/h |
| $kd_{C^{100}}$ | 1.33$\cdot10^{-1}$ | 1/h |
| $kd_{C^{010}}$ | 4.82$\cdot10^{-3}$ | 1/h |
| $kd_{C^{001}}$ | 3.89$\cdot10^{-2}$ | 1/h |
| $kd_{C^{110}}$ | 1.28$\cdot10^{-1}$ | 1/h |
| $kd_{C^{101}}$ | 5.59$\cdot10^{-2}$ | 1/h |
| $kd_{C^{011}}$ | 3.42$\cdot10^{-3}$ | 1/h |
| $kd_{C^{111}}$ | 2.32 | 1/h |
| $V_{f12}$ | 1.88$\cdot10^{3}$ | molecules/h |
| $V_{f13}$ | 1.76$\cdot10^{4}$ | molecules/h |
| $V_{f23}$ | 8.45$\cdot10^{4}$ | molecules/h |
| $V_{f123}$ | 1.64$\cdot10^{4}$ | molecules/h |
| $V_{b12}$ | 5.35$\cdot10^{4}$ | molecules/h |
| $V_{b13}$ | 4.09$\cdot10^{4}$ | molecules/h |
| $V_{b23}$ | 2.77$\cdot10^{3}$ | molecules/h |
| $V_{b123}$ | 2.73$\cdot10^{4}$ | molecules/h |
| $DHHC_{16t}$ | 5.06$\cdot10^{3}$ | molecules |
| $APT_{2t}$ | 5.39$\cdot10^{3}$ | Molecules |

**Supplementary file 3B. Stoichiometric matrix used for stochastic simulations.** In the following table we define the stoichiometry and the directionality of the reactions of the model. Each reaction has a directionality that define which are the reagents and which are the products. In this table each line represents a model reaction, while in the columns we find all the states of the model. For each reaction the states of the model that take part to the reaction as reagents are marked with -1, while the states that participate as products are marked with 1. The matrix that is formed in this way allow to attribute the correct directionality to model reactions during the calculation of the mass balance for each state of the model.

**Supplementary file 3C. Propensity function used for stochastic simulations.** In the first column of the table, each line describes a reaction of the model. To each reaction is associated a rate, in the second column, that describes the probability of that reaction to happen at each time step of the stochastic simulation.

| Reaction | **Propensity** |
| --- | --- |
|  | ** |
|  | ** |
| $C^{000}\to C^{100}$  | ** |
| $C^{000}\to C^{010}$ | ** |
| $C^{000}\to C^{001}$ | ** |
| $C^{000}\to\emptyset$ | ** |
| $C^{100}\to C^{110}$ | ** |
| $C^{100}\to C^{101}$ | ** |
| $C^{100}\to C^{000}$ | ** |
| $C^{100}\to\emptyset$ | ** |
|  | ** |
| $C^{010}\to C^{011}$ | ** |
| $C^{010}\to C^{000}$ | ** |
| $C^{010}\to\emptyset$ | ** |
| $C^{001}\to C^{101}$ | ** |
| $C^{001}\to C^{011}$ | ** |
| $C^{001}\to C^{000}$ |  |
| $C^{001}\to\emptyset$ |  |
| $C^{110}\to C^{111}$ |  |
| $C^{110}\to C^{100}$ |  |
| $C^{110}\to C^{010}$ |  |
| $C^{110}\to\emptyset$ |  |
| $C^{101}\to C^{111}$ |  |
| $C^{101}\to C^{100}$ |  |
| $C^{101}\to C^{001}$ |  |
| $C^{101}\to\emptyset$ |  |
| $C^{011}\to C^{111}$ |  |
| $C^{011}\to C^{010}$ |  |
| $C^{011}\to C^{001}$ |  |
| $C^{011}\to\emptyset$ |  |
| $C^{111}\to C^{110}$ |  |
| $C^{111}\to C^{101}$ |  |
| $C^{111}\to C^{011}$ |  |
| $C^{111}\to\emptyset$ |  |
| $\to U^{*}$ |  |
|  | $kf\cdot U^{*}$ |
|  | $\frac{V_{f1}\cdot C^{000*}}{\frac{Kmf1}{f_{1}}}$ |
| $C^{000*}\to C^{010*}$ | $\frac{V_{f2}\cdot C^{000*}}{\frac{Km_{f2}}{f_{2}}}$ |
|  | $\frac{V_{f3}\cdot C^{000*}}{\frac{Km_{f3}}{f_{3}}}$ |
| $C^{000*}\to\emptyset$ | $kd_{C^{000}}\cdot C^{000*}$ |
| $C^{100*}\to C^{110*}$ | $\frac{V_{f12}\cdot C^{100*}}{\frac{Km_{f2}}{f_{2}}}$ |
|  | $\frac{V_{f13}\cdot C^{100*}}{\frac{Km_{f3}}{f_{3}}}$ |
| $C^{100*}\to C^{000*}$ | $\frac{V_{b1}\cdot C^{100*}}{\frac{Km_{b1}}{b_{1}}}$ |
| $C^{100*}\to\emptyset$ | $kd_{C^{100}}\cdot C^{100*}$ |
| $C^{010*}\to C^{110*}$ | $\frac{V_{f12}\cdot C^{010*}}{\frac{Km_{f1}}{f_{1}}}$ |
| $C^{010*}\to C^{011*}$ | $\frac{V_{f23}\cdot C^{010*}}{\frac{Km_{f3}}{f_{3}}}$ |
| $C^{010*}\to C^{000*}$ | $\frac{V_{b2}\cdot C^{010*}}{\frac{Km_{b2}}{b_{2}}}$ |
| $C^{010*}\to\emptyset$ | $kd_{C^{010}}\cdot C^{010*}$ |
| $C^{001*}\to C^{101*}$ | $\frac{V_{f13}\cdot C^{001*}}{\frac{Km_{f1}}{f_{1}}}$ |
| $C^{001*}\to C^{011*}$ | $\frac{V_{f23}\cdot C^{001*}}{\frac{Km_{f2}}{f_{2}}}$ |
| $C^{001*}\to C^{000*}$ | $\frac{V_{b3}\cdot C^{001*}}{\frac{Km_{b3}}{b_{3}}}$ |
| $C^{001*}\to\emptyset$ | $kd_{C^{001}}\cdot C^{001*}$ |
| $C^{110*}\to C^{111*}$ | $\frac{V_{f123}\cdot C^{110*}}{\frac{Km_{f3}}{f_{3}}}$ |
| $C^{110*}\to C^{100*}$ | $\frac{V_{b12}\cdot C^{110*}}{\frac{Km_{b2}}{b_{2}}}$ |
| $C^{110*}\to C^{010*}$ | $\frac{V_{b12}\cdot C^{110*}}{\frac{Km_{b1}}{b_{1}}}$ |
| $C^{110*}\to\emptyset$ | $kd_{C^{110}}\cdot C^{110*}$ |
| $C^{101*}\to C^{111*}$ | $\frac{V_{f123}\cdot C^{101*}}{\frac{Km_{f2}}{f_{2}}}$ |
| $C^{101*}\to C^{100*}$ | $\frac{V_{b13}\cdot C^{101*}}{\frac{Km_{b3}}{b_{3}}}$ |
| $C^{101*}\to C^{001*}$ | $\frac{V_{b13}\cdot C^{101*}}{\frac{Km_{b1}}{b_{1}}}$ |
| $C^{101*}\to\emptyset$ | $kd_{C^{101}}\cdot C^{101*}$ |
| $C^{011*}\to C^{111*}$ | $\frac{V_{f123}\cdot C^{011*}}{\frac{Km_{f1}}{f_{1}}}$ |
| $C^{011*}\to C^{010*}$ | $\frac{V_{b23}\cdot C^{011*}}{\frac{Km_{b2}}{b_{2}}}$ |
| $C^{011*}\to C^{001*}$ | $\frac{V_{b23}\cdot C^{011*}}{\frac{Km_{b3}}{b_{3}}}$ |
| $C^{011*}\to\emptyset$ | $kd_{C^{011}}\cdot C^{011*}$ |
| $C^{111*}\to C^{110*}$ | $\frac{V_{b123}\cdot C^{111*}}{\frac{Km_{b3}}{b_{3}}}$ |
| $C^{111*}\to C^{101*}$ | $\frac{V_{b123}\cdot C^{111*}}{\frac{Km_{b2}}{b_{2}}}$ |
| $C^{111*}\to C^{011*}$ | $\frac{V_{b123}\cdot C^{111*}}{\frac{Km_{b1}}{b_{1}}}$ |
| $C^{111*}\to\emptyset$ | $kd_{C^{111}}\cdot C^{111*}$ |

Where the competition terms $f$ and $b$ in S1 Table are defined as:

| $f_{1}$ |  |
| --- | --- |
| $f_{2}$ |  |
| $f_{3}$ |  |
| $b_{1}$ |  |
| $b_{2}$ |  |
| $b_{3}$ |  |
