# Supplementary material for: Identification and dynamics of the human ZDHHC16-ZDHHC6 palmitoylation cascade
Source: eLife. 2017 Aug 15;6:e27826. doi: 10.7554/eLife.27826 (PMC5582869; doi:10.7554/eLife.27826)
Supplement: Supplementary file 4. — The table shows the average number of passages of a DHHC6 molecule in the different palmitoylation states when APT2 is silenced. The time spent in each state is also reported. [file elife-27826-supp4.docx]

**Supplementary file 4. Results of stochastic simulation when APT2 is silenced.** The table shows the average number of passages of a DHHC6 molecule in the different palmitoylation states when APT2 is silenced. The time spent in each state is also reported.
